# Online Moral Disengagement: An Examination of the Relationships Between Electronic Communication, Cognitive Empathy, and Antisocial Behavior on the Internet

**DOI:** 10.1177/00332941231216415

**Published:** 2023-11-30

**Authors:** Madison Corkum, N. Will Shead

**Affiliations:** Department of Psychology, 3684 Mount Saint Vincent University, Halifax, NS, Canada

**Keywords:** Cognitive style, relationships & communications, thinking styles, social perceptions, self-perceptions, attributions, moral reasoning, antisocial behavior, sociocultural topics, mental & physical health, decision making, political identification, empathy, cognitive empathy, affective empathy, social interactions, moral disengagement, online behavior, Internet, online communication, anonymity, prosocial behavior

## Abstract

A consequence of the proliferation of online communication is the concerning presence of antisocial behavior observed in virtual spaces. Research suggests the cognitive component of empathy is hindered by features of electronic communication which facilitates antisocial behaviors online. Investigations into how features of online communication inhibit cognitive empathy are lacking, and findings on moral disengagement and antisocial behavior have yet to be integrated with studies on cognitive empathy and electronic communication. The current study explores these relationships. One hundred and three undergraduate students completed several measures including the Online Moral Disengagement Scale, Questionnaire of Cognitive and Affective Empathy, and Online Prosocial and Antisocial Behavior Scale. Results showed a positive correlation between compulsive internet use and online moral disengagement, as well as a negative correlation between cognitive empathy and moral disengagement online. It was hypothesized that online moral disengagement would mediate the relation between cognitive empathy and antisocial behavior online but this mediation was not supported. However, a moderated relationship was revealed between cognitive empathy and moral justification, by liberalism. This moderation can be explored further and built upon by future research to deepen our understanding of how political ideology relates to virtual behavior. Furthermore, the findings concerning components of empathy and moral disengagement, and their role within the perpetration of antisocial conduct online, can inform future research as well as interventions focused on fostering prosocial behavior online and curbing cyberaggression.

## Introduction

Daily interpersonal interactions are becoming increasingly technology-based. From the rapid evolution of social media to online learning in schools, and even virtual training in workplaces, today’s technology is shaping a new way of life. One aspect of this advancement in technology is increased online communication, which, for many, has begun to replace face-to-face interactions. Communication over the internet has become so pervasive that many now consider it to be indispensable (Bjelajac et al., 2022; Larson, 2021). While online communication has immeasurable benefits — such as providing ample opportunities for positive interactions, maintaining connections, and allowing us to benefit in ways that would not otherwise be possible (Lenhart et al., 2015; Nozaki & Mikolajczak, 2022; Rautenbach & Black-Hughes, 2012) — it can also facilitate harmful exchanges. Increased accessibility and use of electronic communication has given rise to new forms of victimization and aggression. Although bullying has been a long-established social issue, and studies on cyberbullying, which refers to repeatedly causing harm through electronic means (Wang et al., 2022) such as sending hurtful or threatening messages, have begun to populate the literature (Guo, 2021; Lowry et al., 2016; Zhao et al., 2022), further research is needed on antisocial behavior online and its associations with socio-cognitive and contextual factors.

A person’s conduct involves personal, behavioral, and environmental determinants (Bandura, 2001). Personal factors, including cognitive, affective, and biological events, as well as behavioral patterns and the external environment all interact to produce psychosocial functioning and behavior. This understanding of reciprocal causation is essential to social cognitive theory (Bandura, 1986), which gives a central role to cognitive and self-reflective processes. People are not simply reactive beings shaped solely by environmental events. Most external influences shape behavior through cognitive processes rather than directly (Bandura, 2001). Cognitive factors help determine which environmental events will be taken in, the meaning given to them, and their emotional impact. According to social cognitive theory, individuals’ decisions concerning their behavior are rooted in the processing of information relating to the behavior and its appraisal. In light of these theoretical considerations, antisocial behavior must be understood by considering the cognitive mechanisms that work to justify these types of action in certain contexts, rather than assuming that it could be solely shaped by environmental influences.

### Empathy and Antisocial Behavior

There is an abundance of research supporting the idea that empathy is crucial to one’s moral development (Barriga et al., 2009; Georgiou et al., 2019; Hawks et al., 2015). Empathy has been studied extensively in relation to antisocial behaviors and developmental theories suggest that empathy is fundamental to the development of prosocial behaviors and the inhibition of antisocial ones (Georgiou et al., 2019). Research suggests that empathic abilities inhibit antisocial conduct — for example, empathy has been shown to be positively correlated with prosocial behaviors (Ang & Goh, 2010; Brazil et al., 2022) and negatively related to antisocial behavior (Brazil et al., 2022; Carré et al., 2013; Hawks et al., 2015) — however, in order to get a deeper understanding of prosocial and antisocial behaviors, we must distinguish between unique components of empathy.

Conceptualizations of empathy have transformed over the years to become considerably more complex. In the 1950s, Carl Rogers described empathy as an ability that allows for the understanding of another individual’s perspective and feelings (Rogers, 1951). This view of empathy, which prevailed for many years, situates it as a solitary, discrete ability. In more recent years, the conceptualization of empathy has evolved to become a multidimensional construct which is now reflected in the literature (Ang & Goh, 2010; Bray et al., 2021). This transition takes into consideration the distinct processes involved in emotion processing and social interactions (Carré et al., 2013). The evolution of the conception of empathy has culminated in an understanding that is based on dual components of empathy: an affective component and a cognitive component (Carré et al., 2013). Affective empathy refers to the ability to experience the emotions that other people are feeling, while cognitive empathy refers to an understanding of another person’s emotional state (Ang & Goh, 2010). Cognitive empathy is the ability to recognize others’ emotions and perspectives (Georgiou et al., 2019). Although the division of empathy into a cognitive and an affective component is widely accepted, some studies propose that there are more than two dimensions of empathy (Derntl et al., 2009). Consequently, there is no single agreed upon model of empathy across the vast body of literature on the topic (Bray et al., 2021). In any case, further research is necessary to investigate the distinct processes involved in empathy and their individual contributions to prosocial and antisocial behavior, particularly in the context of online communication.

Research suggests that empathy depends on bottom-up as well as top-down processing (Carré et al., 2013). The bottom-up processes of affective empathy relate to the sharing of emotions, while the top-down processes of cognitive empathy regulate empathic experience. In the presence of others, there is a tendency to experience similar emotional states — often called “emotional contagion” — which is initiated without conscious reflection as a result of bottom-up processing. In contrast, top-down processing begins with an interpretation of incoming information and empathic experience is regulated by this interpretation. For example, if an individual does not interpret another person’s emotional state accurately, or if the ability for interpretation is hindered, their empathic experience will be modulated.

In their study discriminating between the effects of cognitive and affective empathy on cyberbullying in adolescents, Ang and Goh (2010) reported that cognitive empathy plays a more significant role in aggressive online behavior than the affective component of empathy. They found that those with low levels of cognitive empathy are more likely to carry out cyberbullying behaviors. It makes sense for cognitive empathy to be more salient than affective empathy in the production of reprehensible conduct online considering that the emotional contagion involved in affective empathy occurs in the presence of others. When engaging online, others are not physically present and therefore affective empathy is less likely to occur in these types of interpersonal interactions. In contrast, the top-down processes of cognitive empathy rely on the interpretation of incoming information which dominates online interactions. Interestingly, despite the possibility of emotional contagion occurring in the presence of others, research supports the notion that cognitive empathy plays an even more important role than affective empathy in harmful behavior, not only online but also in “real life.” For example, Georgiou et al. (2019) reported results highlighting the importance of antisocial behaviors being explained by low levels of cognitive empathy. Using a sample consisting of 167 children, a multi-informant approach was used where parents and teachers completed questionnaires about the children. Callous unemotional traits, empathy, conduct problems, and aggression were measured. Their findings pointed towards cognitive empathy being more important than affective empathy in explaining antisocial conduct in children. The results of the study showed that cognitive empathy, but not affective empathy, was negatively associated with antisocial behaviors.

Parlangeli et al. (2019) suggested that cognitive empathy is hindered when interacting online because the greater speed of communication interferes with careful information processing. It has been found that moral emotions such as compassion involve slower information processing than other emotions because they require deeper reflection (Immordino-Yang et al., 2009) and are therefore less likely to occur when engaging with others on the internet. Accurate information processing is necessary for amicable interactions as it allows for an understanding of others’ intent, contextual demands, and decisions around appropriate responses (Smeijers et al., 2020). The Social Information Processing (SIP) model, developed as a framework centered around cognitive contributions to aggression, is described by Smeijers and colleagues as a way to explain how people perceive information and make decisions based on interpretations. According to the SIP model, inaccurate information processing can lead to aggression. This theory provides support for the idea that when information processing is hindered in certain contexts, it can result in aggressive behaviors. A salient feature of communication on the internet is speed. Messages can be sent and comments can be made in an instant, providing little time to process information. Evaluative judgements linked to the initiation of moral emotions are often bypassed in electronic communication due to its speed (Parlangeli et al., 2019). This aspect of the internet impedes individuals’ abilities to employ the cognitive component of empathy and can result in aggressive or offensive acts online.

Numerous avenues of research have shown that anonymity is a major factor in online antisocial behavior (Barlett & Gentile, 2012; Moore et al., 2012; Santana, 2014). Santana (2014) found that 53% of comments posted anonymously were uncivil compared with 29% of comments posted without a hidden identity. Moore et al. (2012) also found that most cyberbullying attacks were anonymous. The anonymity that the internet easily provides is a second characteristic obstructing the cognitive component of empathic abilities (Parlangeli et al., 2019). In addition to avoiding the risk of damaging one’s reputation, the anonymity of electronic communication plays a part in the phenomenon of deindividuation, where levels of self-awareness become decreased in groups, creating environments conducive to antisocial acts (Tang & Fox, 2016). Deindividuation frequently occurs within online communities, where a lack of self-awareness interferes with cognitive processes that are important to the cognitive dimension of empathy. Anonymous electronic environments reduce individuating information about the identity of the users. Indeed, anonymity is considered the principal factor of disinhibited behavior (Lowry et al., 2016) and relates to the online disinhibition effect (Yang et al., 2021). Anonymity allows people to disconnect their conduct online from their identities offline, as though moral cognitive processes are obstructed. This disinhibition effect leads to increased deviance and antisocial behavior online. Kowalski and Limber (2007) highlighted in their study the dangers of online anonymity by stating that people are more inclined to say things on the internet that they would not say to an individual’s face. The perception of anonymity weakens one’s inhibitions. Research shows that aggressor-perceived anonymity is correlated with aggressive online behavior (Barlett & Gentile, 2016). The self-awareness and cognitive reflection that typically occurs in face-to-face transactions has shown to be hindered in anonymous computer-mediated communication, which is detrimental to the cognitive component of empathic abilities.

It is clear that anonymity, as well as the speed of communication, can influence cyberaggression. Evidence suggests that the cognitive component of empathy is hindered by these online characteristics; however, little is known about the relationships involved in how online contexts interact with cognitive empathy to facilitate antisocial behaviors online, particularly in relation to moral disengagement.

### Online Moral Disengagement

Bandura (1999a) was among the first to illuminate the concept of moral disengagement, which has its roots in social cognitive research (Bandura, 1999a, 1999b, 2015). Moral disengagement is a process in which individuals weaken their internal moral standards in order to justify harmful behavior (Wang & Ngai, 2020). It involves cognitive restructuring to situate one’s objectionable behavior as morally acceptable. Reflective cognitive processes that would normally encourage moral behavior are diminished or even absent when individuals morally disengage. Importantly, contextual factors affect an individual’s level of moral disengagement. It may be easier for individuals to morally disengage online due to features of electronic communication like speed and anonymity (Parlangeli et al., 2019). These characteristics of communication on the internet may inhibit cognitive empathy through the facilitation of moral disengagement mechanisms. Therefore, it could be assumed that higher levels of cognitive empathy may be predictive of less online moral disengagement. This would provide additional evidence of the causal relationship between deficits in cognitive empathy and aggressive behavior through features of electronic communication.

With rapid innovations in technology and growing concern around online aggression, research efforts aimed to better understand online antisocial behavior and its correlates are crucial. The results from research examining associations between moral disengagement and cyberbullying has been inconclusive (Wang & Ngai, 2020). Some research found that higher levels of moral disengagement were predictive of increased participation in cyberbullying (Bussey et al., 2015), while other studies found moral disengagement to be less relevant for cyberbullying than for traditional bullying (Bauman & Pero, 2011). Currently, research investigating how online communication interacts with the cognitive component of empathy to produce antisocial behaviors online is scarce, and no integrated studies have examined the potential mediating role of moral disengagement within this relationship.

### Political Ideology and Personality

Research has found that the role of personality is as important as demographic correlates like income and education in predicting political ideology (Gerber et al., 2010). Considerable research has emerged on relationships between personality traits and political ideology, and this research consistently finds that higher openness to experience is associated with support for liberal parties and the left side of the political spectrum (Bergeron & Galipeau, 2021; Blais et al., 2022). People with a high score in openness tend to respond positively to novelty and new ideas (Bergeron & Galipeau, 2021). Blais et al. (2022) found that openness was associated with self-placement on the left of the political spectrum, identification with left-leaning political parties, and less identification with the Conservative Party of Canada. Their results also confirmed that those higher in the honesty-humility factor of the HEXACO model of personality were significantly more likely to place themselves on the left side of the political spectrum, while people who scored higher in narcissism tended to place themselves on the right. Additionally, people who identified with specific liberal political parties scored higher on agreeableness, while those who support a conservative party scored higher on callous-unemotional traits. People high in agreeableness tend to be more altruistic and empathetic (Bergeron & Galipeau, 2021). Importantly, identification with right-wing politics was associated with higher antisocial tendencies such as rule breaking and aggression (Blais et al., 2022). In light of this evidence, it was hypothesized that endorsement in more liberal beliefs would be predictive of less antisocial conduct online.

### The Current Study

The purpose of the current research is to explore how antisocial conduct is facilitated in online settings through interactions with cognitive empathy. It investigates the relationships between cognitive empathy, moral disengagement, liberalism, and conduct on the internet, along with the possibility of moral disengagement acting as a mediating variable between cognitive empathy and antisocial behaviors within electronic communication.

**Figure 1.**
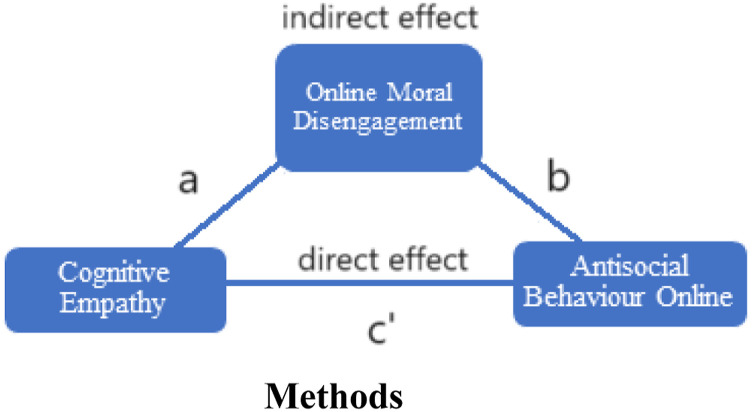
Hypothesized mediation with cognitive empathy as the predictor variable, online moral disengagement as the mediator variable, and antisocial behavior online as the criterion variable.

It was hypothesized that individuals with higher levels of cognitive empathy would show lower levels of online moral disengagement, and that individuals with lower levels of online moral disengagement would report less antisocial behavior online. Therefore, it was predicted that moral disengagement would function as a mediating variable between cognitive empathy and the perpetration of antisocial acts within electronic communication (see Figure 1). It was also hypothesized that individuals with lower levels of online moral disengagement would report more prosocial behavior online. Additionally, because those with liberal political views tend to score higher in agreeableness, honesty-humility, and openness to experience, while those on the right side of the political spectrum show more antisocial behaviors and score higher in narcissism (Blais et al., 2022), we speculated that endorsement in more liberal beliefs would be predictive of less antisocial conduct online.

## Methods

### Participants

A total of 103 undergraduate students residing in Canada participated in the current research (87.4% female). Participants were recruited from Mount Saint Vincent University and ranged in age from 18 to 42 years (*M* = 24.5, *SD* = 7.35). Three attention checks were included in the survey, which were questions that asked participants to select a specific response option in order to ensure the validity of responses. The data of participants who failed more than two attention checks were excluded (*n* = 2). Participants who had missing questionnaire data were also excluded from analyses involving variables associated with those questionnaires.

### Materials

#### Demographic Questionnaire

A brief demographic questionnaire was used to identify the age and gender of participants.

#### IPIP Liberalism Scale

Participants completed the International Personality Item Pool (IPIP) Liberalism scale (Goldberg, 1999), which asks respondents to indicate their agreement with 10 statements, such as “I believe that criminals should receive help rather than punishment”. Responses were made on a 5-point Likert-scale ranging from “strongly disagree” to “strongly agree” creating a range of possible scores from 10 to 50, with higher scores indicating more endorsement of liberal beliefs. Because the IPIP Liberalism Scale was designed to measure a construct similar to the openness domain in Costa and McCrae’s NEO Personality Inventory (NEO-PI-R) (Costa & McCrae, 1992), a primary form of validity is the correlation between the IPIP Liberalism Scale and the NEO-PI-R openness domain on which it was based (International Personality Item Pool, 2022). The 10-item IPIP Liberalism scale has a correlation of .79 with the openness domain of the NEO-PI-R. The corrected correlation coefficient is .91. The Cronbach alpha reliability estimate for the IPIP Liberalism Scale is .86 suggesting good internal reliability.

#### Brief Compulsive Internet Use Scale

Participants completed the CIUS-5 (Lopez-Fernandez et al., 2019) which asks five questions about internet use, such as “Do you go on the internet when you are feeling down?”. Responses were made on a 5-point Likert-scale ranging from “never” to “very often” creating a range of possible scores from 5 to 25, with higher scores indicating more compulsive internet use. The CIUS-5 is a reliable and structurally stable research instrument that has been validated in eight languages (Lopez-Fernandez et al., 2019).

#### Online Moral Disengagement Scale

The Moral Disengagement Scale (MDS) developed by Bandura and his colleagues (1996) is the most commonly used instrument to measure moral disengagement (Gini et al., 2014). The MDS was developed based on eight mechanisms by which moral disengagement occurs (Bandura, 1999a). The scale shows good reliability with a Cronbach’s alpha of .82 (Swann et al., 2017). Additionally, moral disengagement scores were positively associated with multiple reports of aggression and negatively associated with multiple reports of prosocial behavior, indicating sound construct validity. Numerous adapted and revised versions of the MDS have now been developed (Barchia & Bussey, 2011; Gini et al., 2014; Pelton et al., 2004). Because of the context-specific nature of moral disengagement, measures of the construct that are contextualized to antisocial behaviors, such as cyberbullying, are more strongly related to these behaviors than a moral disengagement scale measuring a wide range of objectionable behaviors (Quinn & Bussey, 2015). Accordingly, this study used an author-adapted version of the MDS so that the items would reflect antisocial conduct during electronic communication. The 19-item author-adapted version of the MDS asked participants to indicate their level of agreement with statements such as “People who get mistreated on the internet usually do things that deserve it” and was scored on a 5-point Likert-scale from “strongly agree” to “strongly disagree” creating a range of possible scores from 19 to 95, with higher scores indicating lower levels of moral disengagement. Based on the current sample, a reliability analysis showed this adapted version of the MDS had good internal reliability with a Cronbach’s alpha of .87.

#### Questionnaire of Cognitive and Affective Empathy

Empathy was measured using the Questionnaire of Cognitive and Affective Empathy (QCAE) (Reniers et al., 2011). The scale consists of 31 items — 19 items measuring cognitive empathy and 12 items measuring affective empathy. It asks participants to indicate their level of agreement with statements such as “I find it easy to put myself in somebody else’s shoes” (cognitive empathy) and “I get very upset when I see someone cry” (affective empathy), and is scored on a 4-point Likert-scale from “strongly disagree” to “strongly agree”. This creates a range of possible scores from 31 to 124, with higher scores indicating higher levels of empathy. The QCAE has been shown to have good reliability (Reniers et al., 2011). The cognitive and affective empathy scores on the QCAE and the Basic Empathy Scale (another popular measure of empathy) show strong positive correlations, indicating good convergent validity. Additionally, strong evidence of construct validity was shown through significant differences between cognitive and affective empathy in relation to theoretically relevant measures such as questionnaires assessing impulsivity, empathic anger, and aggression. Furthermore, the items of the QCAE were derived from established and validated questionnaires, which adds to their strengths.

#### Online Prosocial and Antisocial Behavior Scale

An adapted version of Erreygers et al.’s (2017) scale measuring engagement in online prosocial and antisocial behavior was used. The online antisocial behavior items of the scale were drawn by Erreygers and colleagues from the European Cyberbullying Intervention Project Questionnaire (ECIPQ; Del Rey et al., 2015) which was shown to have excellent overall reliability (Del Rey et al., 2015). Erreygers et al. (2017) created the items of the scale measuring online prosocial behavior by adapting items used by Wright and Li (2011) and adding items based on measures of offline prosocial behavior. The online prosocial behavior items were later made into an independent scale called the Online Prosocial Behavior Scale (OPBS), validated using the same sample (Erreygers et al., 2018). The subscales of the OPBS had significantly positive correlations with offline prosocial behavior and use of digital media, providing support for the convergent validity of the OPBS. For the online prosocial behavior items, participants rated their frequency of online prosocial behavior by selecting a response on a 5-point Likert-scale, ranging from “never” to “every day”, indicating how often in the past month they engaged in each behavior. Ten statements were used to measure online prosocial conduct, such as “Compliment or congratulate someone”. This creates a range of possible scores from 10 to 50, with higher scores indicating more frequent prosocial behavior. Similarly, for the online antisocial behavior items of the scale, participants rated their frequency of online antisocial behavior by selecting a response from a 5-point Likert-scale, ranging from “never” to “almost every day”, indicating how many times in the past month they engaged in each behavior. Eleven statements measured online antisocial conduct, such as “I posted personal information about someone online” creating a range of possible scores from 11 to 55, with higher scores indicating more frequent antisocial behavior.

### Procedure

Participants were recruited from Mount Saint Vincent University through a research participation management system, course management platforms (i.e., Moodle), and posts made on social media pages. Participants received course credit at the discretion of their instructors for taking part in the study.

Participants were directed to LimeSurvey, an open-source online survey platform, where they read over a consent form. After providing informed consent, participants answered two brief demographic questions, before being asked to complete five questionnaires: the IPIP Liberalism Scale, the Brief Compulsive internet Use Scale (CIUS-5), the Online Moral Disengagement Scale, the Questionnaire of Cognitive and Affective Empathy (QCAE), and the Online Prosocial and Antisocial Behavior Scale. At the end of the questionnaires, participants were taken to an end-of-survey page.

### Ethical Considerations

This study was cleared by the Mount Saint Vincent University Research Ethics Board and respects the *Tri-Council Policy Statement: Ethical Conduct for Research Involving Humans*. Written informed consent was obtained for all participants. The study adhered to the principles of the Declaration of Helsinki.

## Statistical Analysis

To test the hypothesized correlations and mediations, SPSS Statistics 28 software (IBM Corp, 2021) and the PROCESS macro (Model 4) developed by Hayes (2013) were used to estimate all parameters. Two-tailed Pearson correlations were used. Indirect effects were examined using 95% bias-corrected bootstrapped confidence intervals (with 5000 resamples).

## Results

Bivariate correlations were calculated between the relevant measures. Descriptive statistics and correlations between all variables are reported in Table 1.

**Table 1.** Descriptive Statistics and Bivariate Correlations Between Measures.

Variable	M(SD)	1	2	3	4	5	6	7
1. CIUS	10.17 (4.53)	—						
2. MDS	57.45 (10.96)	−.244*	—					
3. IPIP	22.58 (6.65)	−.241*	.009	—				
4. OPBS	37.55 (7.17)	.246*	−.177	−.312**	—			
5. OABS	1.73 (3.60)	.165	−.252*	.001	.141	—		
6. Cog. Emp	54.58 (5.57)	−.034	.265*	−.178	.462**	−.353**	—	
7. Aff. Emp	33.92 (3.13)	.087	.038	−.219*	.457**	−.162	.604**	—

CIUS = Compulsive Internet Use Scale; MDS = Online Moral Disengagement Scale; IPIP = International Personality Item Pool Liberalism Scale; OPBS = Online Prosocial Behavior Scale; OABS = Online Antisocial Behavior Scale; Cog. Emp = Cognitive Empathy Subscale of Questionnaire of Cognitive and Affective Empathy; Aff. Emp = Affective Empathy Subscale of Questionnaire of Cognitive and Affective Empathy.

*p < .05; **p < .01.

### Correlation Analyses

In line with expectations, individuals with higher levels of cognitive empathy showed lower levels of online moral disengagement (higher scores on MDS reflected lower online moral disengagement). The relationship between cognitive empathy and online moral disengagement was statistically significant, *r* (86) = .265, *p* = .012. The correlation between affective empathy and online moral disengagement was not statistically significant, *r* (92) = .038, *p* = .715, indicating an important distinction between the cognitive and affective components of empathy in relation to online moral disengagement.

Also as predicted, the relationship between online moral disengagement and antisocial behavior online was statistically significant, *r* (94) = −.252, *p* = .013. Individuals with lower levels of online moral disengagement reported less antisocial behavior online. This points towards moral disengagement playing an important role not only in direct objectionable interpersonal interactions but also in antisocial conduct in virtual settings. Our hypothesis that individuals with lower levels of online moral disengagement would report more prosocial behavior online, however, was not confirmed. The relationship between online moral disengagement and online prosocial behavior was not statistically significant, *r* (93) = −.177, *p* = .086. This finding is interesting, as it suggests that online prosocial and antisocial behavior are not simply two poles of a construct, but rather two distinct constructs.

Correlation analyses conducted between liberalism and online conduct showed that liberalism was not related to online antisocial behavior, *r* (91) = .001, *p* = .991, but was correlated with online prosocial behavior, *r* (90) = −.312, *p* = .002. This finding also highlights the distinction between the constructs of prosocial and antisocial behavior online.

Additionally, it was found that there is a significant relationship between compulsive internet use (as measured by the CIUS-5) and online moral disengagement, *r* (97) = −.244, *p* = .015. Those whose internet use was more compulsive tended to employ more moral disengagement online.

As previous research suggested, cognitive empathy was correlated with online antisocial behavior, *r* (88) = −.353, *p* = < .001, while the relationship between affective empathy and antisocial behavior online was not statistically significant, *r* (92) = −.162, *p* = .118.

### Mediation Analysis

A mediation analysis was conducted to examine the hypothesis that online moral disengagement plays a mediating role between cognitive empathy and antisocial behavior online. The results of this analysis are reported in Table 2. The paths noted in the table correspond to those depicted in Figure 1. There was a significant relation between the predictor variable (i.e., cognitive empathy) and the criterion variable (i.e., antisocial behavior online), *r* (86) = .352, *p* = .001*.* There was also a significant relation between the predictor variable (i.e., cognitive empathy) and the hypothesized mediating variable (i.e., online moral disengagement), *r* (86) = .265, *p* = .013. However, there was not a statistically significant indirect effect of cognitive empathy on antisocial behavior online via online moral disengagement, *indirect effect* = −.027, 95% CI [−.090, .002].

**Table 2.** Mediation Results.

Path	*b*	SE	95% CI
c path (X to Y)	−.230	.066	[−.361, −.099]
a path (X to M)	.535	.210	[.118, .951]
b path (M to Y)	−.050	.034	[−.117, .017]
c’ path	−.203	.068	[−.338, −.068]
c path = cognitive empathy → antisocial behavior online (total effect of cognitive empathy on antisocial behavior online)			
a path = cognitive empathy → online moral disengagement			
b path = online moral disengagement → antisocial behavior online			
c’ path = cognitive empathy → antisocial behavior online (direct effect of cognitive empathy on antisocial behavior online, controlled for online moral disengagement)			

Model summary	R	*R* ^2^	MSE	F	df1	df2	*p*
	.352	.124	11.68	12.15	1	86	.0008

### Moderation Analysis

**Figure 2.**
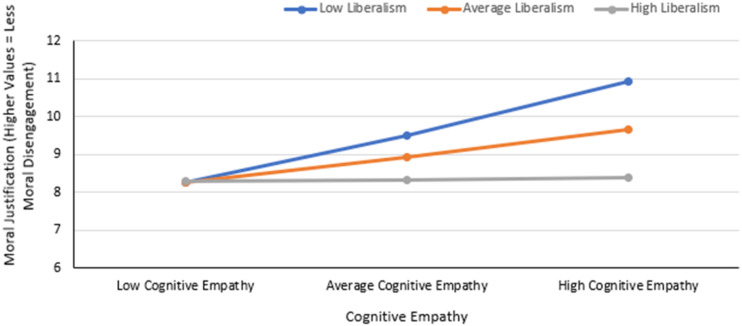
The moderated relationship between cognitive empathy and moral justification by liberalism.

**Figure 3.**
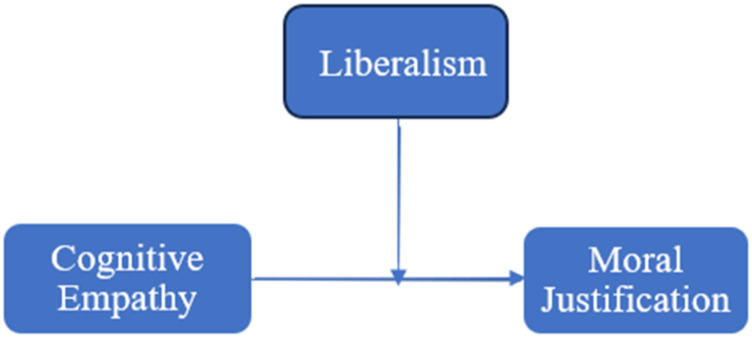
Conceptual model of the moderation with cognitive empathy as the predictor variable, liberalism as the moderator variable, and moral justification as the criterion variable.

Although the mediation analysis did not show an indirect effect of cognitive empathy on antisocial behavior online via online moral disengagement, the analysis showed a significant relation between cognitive empathy and online moral disengagement. It was postulated that this relation might depend on one’s level of liberalism. A post-hoc analysis was done using the PROCESS macro (Hayes, 2013) Model 1, which takes into account the effect of putative moderators, rather than including mediating variables. The model summary for this analysis is reported in Table 3. This analysis revealed a moderated relationship between cognitive empathy and the moral justification sub-scale of online moral disengagement, by liberalism, ∆*R*^2^ = .052, ∆F (1, 83) = 5.06, *p* = .027 (see Figure 2). The relation between cognitive empathy and moral justification depended on liberalism (see Figure 3) — there is a strong relation among those low in liberalism, a moderate relation among those average in liberalism, and almost no relation among those high in liberalism.

**Table 3.** Moderation Model Summary.

Model summary	R	*R* ^2^	MSE	F	df1	df2	*p*
	.420	.176	5.36	5.78	3	81	.0012

## Discussion

The internet provides a social space that is steadily growing. The ease of contact and communication afforded by the internet is beneficial in myriad ways, however, it also allows much of the antisocial behavior that exists within interpersonal interactions to be translated to online settings. Studies in recent years have begun to reveal the reality of not only a strong presence of antisocial conduct in computer-mediated communication, but also the facilitation of it (Ang & Goh, 2010; Barlett et al., 2016; Parlangeli et al., 2019). For example, much research has established the hostility of online gaming environments (Tang & Fox, 2016). Features of online gaming spaces such as anonymity and limited nonverbal cues contribute to the facilitation of conflict, harassment, and antisocial interactions online. Where online spaces provide perceived anonymity, users experience deindividuation, or a loss of a sense of self, influencing disinhibited behavior. Social media has also been commonly used as a medium for threats, stalking, cyberbullying, and harassment, sometimes having extreme consequences on victims from mental health issues to suicide (Kumar & Bhat, 2022). This concerning appearance of hostility and harassment is not restricted to one area of computer-mediated communication, such as gaming or social media. Although harmful conduct is certainly prevalent in these areas, antisocial behavior extends to every corner of the social space provided by the internet, even reaching digital news media. A recent newspaper article in the National Post highlights instances of online attacks and threats against journalists (Roberts, 2022). Examples included disturbing emails and direct messages received by reporters. A poll of online harassment of members of the media revealed that about two-thirds of journalists have experienced harassment online, and one in five face it weekly or worse. This epidemic of cyberaggression and antisocial conduct in virtual spaces needs careful attention and must be addressed in order to stop its proliferation. The current research explores how antisocial conduct is facilitated in electronic means of communication through interactions with cognitive empathy. It examines the relationships between cognitive empathy, moral disengagement, and conduct on the internet.

Empathy is a multidimensional construct comprised of a cognitive and affective component. It is a pivotal factor in aggressive and antisocial behavior, both in person and in online contexts. Cognitive empathy — the component of empathic abilities that refers to one’s capacity to interpret and understand another’s mental state (Bray et al., 2021) — has been shown to play a relatively prominent role in objectionable behavior online, compared to the affective component of empathy (Ang & Goh, 2010; Parlangeli et al., 2019). Georgiou et al. (2019) reported findings showing that cognitive empathy, but not affective empathy, was negatively associated with antisocial behaviors. The current research extends these findings to online contexts, as cognitive empathy was found to be negatively associated with online antisocial behavior, while there was no significant relationship between affective empathy and antisocial behavior online.

Previous research suggests that features of online contexts facilitate cyberaggression and antisocial conduct on the internet by interfering with the cognitive component of empathy (Ang & Goh, 2010; Parlangeli et al., 2019). Characteristics of online communication like speed and anonymity work to obstruct cognitive empathy, resulting in a heightened presence of antisocial conduct in the context of computer-mediated communication (Parlangeli et al., 2019). Shielded from judgment and repercussions by the anonymity of the internet, individuals’ tendency towards cognitive reflection is decreased, often resulting in negative interpersonal exchanges that would have been avoided face-to-face. The phenomenon of deindividuation and the online disinhibition effect have been studied extensively in relation to online anonymity and have been shown to have a strong influence on the perpetration of antisocial acts in computer-mediated communication (Lowry et al., 2016; Tang & Fox, 2016; Yang et al., 2021). The quick pace of online communication also obstructs one’s ability to reflect on potential behaviors in advance. Messages can be sent back and forth almost instantly, and while advantageous in many situations, this can be dangerous. Cognitive reflection is pertinent to the cognitive dimension of empathy, which involves top-down processes that rely on interpretation and reflection (Carré et al., 2013). These top-down processes of cognitive empathy regulate empathic experience, making them salient in relation to the perpetration of antisocial behavior. Although research has provided evidence that cognitive empathy is hindered in online contexts, further research needs to be conducted on the interactions between cognitive empathy and the perpetration of antisocial conduct online. In particular, there is a lack of research around these relationships in relation to moral disengagement online. The current research addresses this gap in the literature.

Moral disengagement is a process that permits the selective use of cognitive mechanisms that serve to justify reprehensible actions (Bussey et al., 2015). It involves self-regulatory processes that allow one to selectively activate and disengage their internal moral standards. In doing so, this enables an individual to behave objectionably while maintaining the same moral standards. Self-sanctions are disengaged through mechanisms of moral disengagement. When engaging in harmful behavior, moral disengagement allows individuals who generally disapprove of this type of conduct to behave antisocially by selectively disengaging their moral standards in order not to lose self-regard. Findings from the current study that higher levels of cognitive empathy were predictive of less online moral disengagement strengthen the existing evidence for deficits in cognitive empathy creating conditions conducive to antisocial behavior through features of electronic communication. The relationship between cognitive empathy and online moral disengagement was significant, yet the correlation between affective empathy and online moral disengagement was not. These contrary findings indicate an important distinction between the cognitive and affective components of empathy in relation to online moral disengagement.

The results from previous research examining associations between moral disengagement and cyberaggression has been inconclusive (Wang & Ngai, 2020). While some research found that higher levels of moral disengagement were predictive of increased participation in cyberbullying (Bussey et al., 2015), other studies found moral disengagement to be less relevant for cyberbullying than for traditional bullying (Bauman & Pero, 2011). The current study found that individuals with lower levels of online moral disengagement reported less antisocial behavior online, indicating that moral disengagement is relevant not only in person, but also in the context of online interaction. Interestingly, our prediction that those with lower levels of online moral disengagement would show more *prosocial* behavior online was not confirmed, suggesting that rather than falling on opposite ends of the same dimension, prosocial and antisocial behavior may be separate and distinct constructs. Additionally, the current study found that liberalism was not related to online antisocial behavior but *was* correlated with online prosocial behavior, again highlighting a distinction between the constructs of prosocial and antisocial behavior online. Hennigan and Cohn (2022) express that because moral traits, such as empathy and proneness to guilt, are associated with prosocial and antisocial behaviors in opposite directions, it suggests that prosocial and antisocial behaviors are opposite ends of the same dimension. However, prosocial and antisocial behaviors appear to be driven by unrelated neural processes. Additionally, recent studies have shown that individuals can simultaneously demonstrate prosocial and antisocial behaviors which can work together in achieving a goal (Basurto et al., 2016; Bodin et al., 2020; Prediger et al., 2014). These studies suggest that prosocial and antisocial behaviors are separate constructs and not a singular dimension, and the current study provides evidence that this applies to online contexts as well. Further research on prosocial and antisocial behavior as separate dimensions can provide a closer look at correlates of each, and aid in the development of intervention programs targeting antisocial conduct and promoting prosocial behaviors, both in social spaces provided by the internet as well as in schools, workplaces, and communities.

The current research found that those whose internet use was more compulsive tended to employ more online moral disengagement. In a world where children are being introduced to numerous methods of interaction in online spaces at increasingly younger ages, it is important to draw attention to the dangers of compulsive internet use. With the rapid development and expansion of the social space provided by the internet, it can be difficult not to become immersed in it, but research findings highlight the importance of limiting our use of the internet to avoid its pitfalls. Findings from the current study on compulsive internet use suggest that as our internet usage becomes more compulsive, we are at a higher risk for disengaging our morals while online and behaving in an antisocial manner.

It was hypothesized that moral disengagement would function as a mediating variable between cognitive empathy and the perpetration of antisocial acts within electronic communication. The mediation analysis did not demonstrate an indirect effect of cognitive empathy on antisocial behavior online via online moral disengagement. Further research in this area is warranted, as mediation analyses can provide important information on the explanatory mechanisms involved in the relationship between cognitive empathy and the perpetration of offensive acts online. Additionally, because the current study used a sample of undergraduate students, this area of research may benefit from further studies using non-university-based community and clinical samples.

A post-hoc analysis revealed that the relationship between cognitive empathy and the moral justification sub-scale of online moral disengagement was moderated by liberalism. As liberalism decreased, the relationship between cognitive empathy and moral justification increased. Moral justification is one of the mechanisms by which moral disengagement occurs (Bandura, 1999a). It is a set of disengagement practices in which an individual justifies to themself their detrimental conduct. Through moral justification, people justify to themselves the morality of their actions before engaging in harmful behavior. This process operates on the construal of the behavior itself. Cognitive reconstruction can situate injurious conduct as acceptable, both personally and socially, by portraying it as serving social or moral purposes. The current findings show that among those with high liberalism, there was no relationship between cognitive empathy and moral justification. However, the relationship between cognitive empathy and moral justification was moderate among those with average liberalism and even more pronounced among those with low liberalism. In other words, among those who espouse viewpoints that emphasize equality and individual rights, one’s use of cognitive empathy was not related to their tendency to morally justify their online actions. A growing body of evidence exists for liberal political views and discourse being connected to empathy, particularly cognitive empathy (Hodson et al., 2019). Hodson and colleagues (2019) share the common perspective that empathy involves two aspects, an affective component (i.e., empathic concern) and a cognitive component (i.e., perspective-taking). They explain how, empirically, those who endorse right-wing political ideologies are perceived as less empathic than those who are left-wing, with self-reports confirming these relationships. Similarly, trends around right-wing constructs have also been observed — higher social dominance orientation is associated with lower cognitive empathy (perspective taking), and those higher in right-wing authoritarianism score lower in empathy. Hodson and colleagues (2019) themselves found that differences in cognitive empathy characterize left-right gaps. Politically right-leaning states scored consistently lower in perspective taking (cognitive empathy), while their findings for empathic concern (affective empathy) were less consistent. They suggest that political differences may have a lot to do with relating to others. A possible explanation for the relationship between cognitive empathy and moral justification depending on liberalism could be that when high levels of liberalism are involved, cognitive empathy is eclipsed by liberalism, due to their entangled nature. It is possible that for those low in liberalism (i.e., openness, tolerance, respect for views different from their own), cognitive empathy is an important factor in the process of moral justification (i.e., cognitive reconstruction of objectionable actions), while for those high in liberalism, these traits supersede the role of cognitive empathy within this relationship. In other words, cognitive empathy may become less important in its relation to moral justification as endorsement in liberalist beliefs increase because for those with strong liberal beliefs, these beliefs take the place of cognitive empathy in combatting the use of moral justification. Among those low in liberalism, cognitive empathy works against the disengagement practice of moral justification, but because of the related nature of cognitive empathy and liberalism, it may be the liberalist beliefs that are salient in combatting the use of moral justification among those high in liberalism.

### Limitations

A potential limitation of this study is the use of self-report questionnaires to collect data for empirical analyses. This approach can lead to methodological issues such as self-report bias. To minimize such biases, survey respondents were assured that their identity and responses to all survey questions would remain anonymous. Nonetheless, further research should use different methods of gathering data in addition to self-report measures. Also, because the current study used a sample of undergraduate students, potentially limiting the representativeness of the results, this area of research may benefit from future studies using non-university-based samples.

It should be noted that the hypothesized model of mediation depicted in Figure 1 implies causal relationships among the variables even though the mediation analysis only provides information about the associations among the variables. That is, the conceptual model suggests that cognitive empathy influences online moral disengagement which, in turn, influences antisocial behavior online. However, a limitation of the PROCESS macro is that it only provides information about the relations among these variables and not about the causality of these relationships. Despite these limitations, the current study draws attention to important relations among variables that may help explain why people engage in antisocial behavior online and offers valuable avenues for further research in this area.

### Conclusion

Growing concern around cyberaggression is warranted by the pervasive presence of harmful conduct on the internet and is exacerbated by rapid innovations in technology and across different forms of electronic communication. As more relationships are being formed and maintained online, the importance of safe cyberspaces and internet culture is only increasing. Given the emerging research on relationships between personality traits and political ideology, much of which suggests that political differences are involved in how we relate to others (Hodson et al., 2019), information on how political ideologies are related to the production of antisocial behavior online is necessary if we wish to cultivate healthy online environments within the expanding social space of the internet. The moderated relationship found in the current study can be further explored in future research to deepen our understanding of how political ideology relates to online conduct. Research efforts focused on the prevention and intervention of injurious online behavior are more important than ever amid an epidemic of cyberaggression. Results from the current study support previous research on the role of cognitive empathy in objectionable online behavior and extend Georgiou et al.’s (2019) findings on cognitive empathy to online contexts. Additionally, while it was found that online moral disengagement had no relationship with affective empathy, it did have a significant association with cognitive empathy. Seeing that cognitive empathy plays an important role in reprehensible conduct within electronic communication, interventions focused on enhancing cognitive empathy over the internet may work to diminish the presence of antisocial behavior online. Also contributing to the literature is evidence from the current study that moral disengagement is relevant not only in person, but also in the context of online interaction. This finding is important to note when conducting research on moral disengagement, as we are just beginning to uncover the similarities and differences in how moral disengagement occurs in online and offline contexts. For example, it was found that as our internet usage becomes more compulsive, we are at a higher risk for disengaging our morals while online. This finding about moral disengagement appears to be unique to online contexts. Research aimed to better understand online antisocial behavior and its correlates are crucial to endeavours to confront the growing problem of cyberaggression. Thus, the current study addressed a gap in the literature concerning the integration of studies on moral disengagement and antisocial behavior with those on cognitive empathy and electronic communication. Findings concerning components of empathy, moral disengagement, and political ideology, and their role in perpetuating antisocial online conduct can inform future research, as well as interventions focused on fostering prosocial behavior online and constraining the threatening social issue of detrimental online behavior.

## Data Availability

The participants of this study did not give written consent for their data to be shared publicly so supporting data is not available.
